# The 10th Anniversary of *Toxics*: Recent Advances in Toxicology Research

**DOI:** 10.3390/toxics12080538

**Published:** 2024-07-26

**Authors:** Demetrio Raldúa, Xiaojun Luo

**Affiliations:** 1 Department Environmental Chemistry, IDAEA-CSIC, Jordi Girona 18, 08034 Barcelona, Spain; 2 State Key Laboratory of Organic Geochemistry, Guangzhou Institute of Geochemistry, Chinese Academy of Sciences, Guangzhou 510640, China

## 1. Introduction

Last year (2023) was the tenth anniversary of *Toxics*. To commemorate this milestone, we launched this Special Issue entitled “*The 10th anniversary of Toxics*”. This Special Issue attracted the interest of a large number of scientific researchers, and 19 papers were finally published. In this Special Issue there are 6 reviews and 13 research articles, covering a wide range of chemicals of environmental concern, from legacy pollutants, such as heavy metals, polychlorinated biphenyls (PCBs), and bisphenol A (BPA), to emerging pollutants such as perfluorinated compounds, microplastics, pharmaceuticals, and pesticides. The research contents include analysis of the adverse effects and toxic mechanisms of these chemicals, the development of new detection methods, biological and human exposure monitoring, theoretical prediction of compound toxicity, and environmental management of chemicals. These studies reflected the current research hotspots and important environmental concerns, and also provide unique proposals for future research. We would like to express our heartfelt gratitude to the authors of these studies. It is their contributions that have made this Special Issue a great success.

## 2. An Overview of Published Articles

Per- and polyfluoroalkyl substances (PFAS), a group of persistent environmental endocrine-disrupting chemicals (EDCs), have become a growing concern in recent years due to increasing evidence of their potentially causing adverse effects in animals and humans [1,2]. Three articles in this Special Issue focus on the association between PFAS exposure and human diseases. Alsen et al. (Contribution 1) evaluated the potential correlation between PFAS levels in drinking water and thyroid cancer incidence in the US and found a statistically significant correlation between perfluorooctanoic acid, perfluorononanoic acid, and thyroid cancer incidence. Boafo et al. (Contribution 2) investigated the association of PFAS with allostatic load (AL), an index of chronic physiological stress, using data from the National Health and Nutrition Examination Survey 2007–2014. The results indicated that PFAS exposure increased the likelihood of higher AL among those with persistent herpes simplex virus infections. Jiang and Guo (Contribution 3) summarized reported evidence regarding the involvement of the gut microbiome and liver inflammation in the onset and exacerbation of autism spectrum disorder symptoms. The authors also discussed potential interactions between PFAS and acetaminophen in terms of the gut–liver–brain axis and signaling pathways that may contribute to neurological diseases. They proposed a new perspective on the exacerbation of autism spectrum disorder by PFAS through the gut–liver–brain axis.

The presence of microplastics (MP) and pharmaceuticals in the environment poses a growing threat to all life forms [3,4]. Weis and Alava (Contribution 4) conducted a comprehensive review on the toxic compounds added into plastics and those adsorbed to the MPs from environment. This review also summarizes the latest progress in the MP research field, such as the role of biofilm in affecting the transfer of contaminants on/to and from MPs, chemicals transferring from MPs into organisms, the effects of aging, weathering, and leachates, as well as toxicity studies on MP leachates. The information presented in this review supports the consideration of MPs as “toxic pollutants”. Papaioannou et al.’s (Contribution 5) article review is focused on pharmaceutical pollution. They first presented an overview of the pharmaceutical pollution in aquatic ecosystems, including sources, fate, treatment methodologies, and their impacts. Then, they comprehensively introduced the eDNA metabarcoding, including technical details, as a robust bioindicator method for evaluating pharmaceuticals’ impacts on organisms.

Pesticides are a major concern in the field of environmental toxicology due to their potential impact on ecosystems and human health. Two articles described the pollution related to pesticides in this Special Issue. Iohanna et al. (Contribution 6) developed a simple and cost-effective methodology to detect and quantify the herbicides glufosinate and glyphosate, as well as the degradation product of the latter, aminomethylphosphonic acid (AMPA), in human plasma and urine samples. By using the proposed methodology, they report for the first time the presence of glufosinate in human matrices, information that can be used to establish baseline values for future surveillance systems. Campanale et al. (Contribution 7) reviewed the latest advancements in another group of pesticides, the dithiocarbamates. They provided a comprehensive survey on all aspects of dithiocarbamates, including analytical methods, their occurrence in plants, animals, and humans, environmental fate, toxicity, and their health effects on humans.

Polycyclic aromatic hydrocarbons (PAHs) are ubiquitous pollutants which come from combustion processes. Benzo[a]pyrene (BaP), as a strong carcinogenic compound, has been widely studied [5,6]. Two articles in this Special Issue focus on the toxicology of BaP. Feltner et al. (Contribution 8) investigate the genetic difference that could affect susceptibility following development exposure to BaP. They found a significantly and surprisingly high neonatal lethality in offspring born to Ahr Cyp1b1(−/−) knockout mice and there was a significant effect of BaP on growth rates in surviving pups, with lower weights observed from postnatal day 7 to 21. They also measured the concentrations of BaP and their metabolites in dams and pups, finding that the concentration of BaP and their metabolites varied with genetic variation in the AHR and CYP 1 genes. Alesci et al. (Contribution 9) evaluated hemocytes in the mantle and gills of *M. galloprovincialis* as biomarkers of thiacloprid and BaP pollution and investigated their possible synergistic effects using mono- and polyclonal anti-Toll-like receptor 2 (anti-TLR2) and anti-inducible nitric oxide synthetase (anti-iNOS) antibodies. Changes in the mantle and gills were evidenced at the histological level. A significant increase in the number of hemocytes was found in the exposure group, and a synergistic effect between the two compounds was found for this endpoint. Thiacloprid and BaP exhibited also a synergic effect for increasing superoxide dismutase and catalase activities. This study provides useful insights to improve the understanding of the roles of TLR2 and iNOS in the internal defense systems of bivalves.

The article by Spiegelhoff et al. (Contribution 10) aimed to understand whether developmental exposure to PCB mixture altered prostate morphology and function in the young adult offspring of mice. The results suggested that developmental exposure to PCB can influence prostate wet weight and prostate/bladder morphology but does not promote prostate enlargement. Whether these changes remain throughout adult life and how they contribute to voiding function in animal models and humans deserves further research. The research from Thorpe et al. (Contribution 11) delves into the pulmonary effects of sub-chronic third-hand e-vapor exposure in a murine model. Mice exposed to third-hand e-vapor with nicotine had reduced bronchial responsiveness to provocation, increased epithelial thickening in large airways, increased epithelial layers in small airways, alveolar enlargement, and increased small airway collagen deposition compared to controls. The results of this study highlighted the need for greater public awareness surrounding the dangers of third-hand exposure to e-cigarette vapor.

Environmental and/or occupational exposure to heavy metals are a major risk, contributing to the development of chronic diseases [7]. Huang et al. (Contribution 12) investigated the toxicity of titanium dioxide (TiO_2_) nanoparticles in the *Caenorhabditis elegans* model. They found that exposure to TiO_2_ NPs has no influence on survival rate and body length. However, they found that it reduced the reproduction, locomotion, and longevity of the nematodes and commercially available TiO_2_ NPs possibly caused more toxicity or genotoxicity in this animal model than synthetically prepared TiO_2_ NPs. The work of Mercado et al. (Contribution 13) assessed the toxicity of three heavy metal (Cr, Cu, and Cd) on three organisms with different trophic levels. The results of the experiments highlighted species-specific sensitivity to heavy metal exposure and further supported previous studies that have suggested the jellyfish *Aurelia aurita* as a suitable model organism for ecotoxicity testing. Burger et al. (Contribution 14) reported levels of heavy metals in horseshoe crab eggs collected from Delaware Bay. The difference in heavy metal content between surface eggs and clutches’ eggs (laid beneath the sand), locational differences, the temporal trend of heavy metals, and the potential effects of heavy metals on horseshoe eggs and shorebirds were also discussed in the study. Since a direct correlation was found between the metal content in the eggs and the blood of shorebirds, the author urges that metals in crab eggs should continue to be monitored as a potential early warning sign of any future effects in the study area. Martins et al. (Contribution 15) reviewed the literature that has associated metabolic syndrome (MetS) with heavy metals exposure. Toxic metals such as mercury, lead, and cadmium may contribute to the development of MetS by altering oxidative stress, IL-6 signaling, apoptosis, altered lipoprotein metabolism, fluid shear stress and atherosclerosis, and other mechanisms. The details on the known and potential roles of heavy metals in MetS etiology as well as the potential targeted pathways that are associated with MetS were also provided in this review article. Nevertheless, the mechanisms by which toxic compounds trigger MetS are not yet fully understood. The authors describe how new approaches involving proteomic and transcriptome analysis, as well as bioinformatic tools, may help bring about an understanding of the involvement of heavy metals and metalloids in MetS.

The computational method offers a highly efficient and cost-effective alternative to traditional experimental approaches for assessing the toxicity of chemicals [8,9]. To address the challenge of assessing the carcinogenic potential of hazardous chemical mixtures, Limbu et al. (Contribution 16) propose a novel framework which utilizes a hybrid neural network (HNN) integrated into a machine learning framework. This method has exceptional predictive power in prioritizing carcinogenic chemical mixtures, offering a novel alternative for dose-dependent carcinogen prediction.

Tsai et al. (Contribution 17) surveyed the up-to-date information about the production and environmental distribution of BPA in Taiwan over the past decade. The majority of the BPA concentrations were far below the acceptable risks but some high BPA concentrations in Taiwanese rivers, especially in the rivers by industrial wastewater discharge, may pose an adverse threat to the aquatic environment and human health via the ecological and food chains. They also analyzed the regulatory strategies and countermeasures for managing the environmental risks of BPA undertaken by the Taiwan government. To echo the international actions on environmental endocrine disruption in recent years, some countermeasures were further recommended in the review.

The last two articles report on the greenhouse gas trends, and the use of a natural flower product as a chemotherapeutic adjuvant in a mouse tumor model. Bărbulescu (Contribution 18) analyzed the temporal trend of greenhouse gases in EU countries and their associates from 1990 to 2021. The following two subperiods are determined: before 2002, with a logarithmic trend, and after 2003, with a linear trend. Overall, the trend of the total GHGs series during 1990–2021 is decreasing. Regarding each country, 17 countries show a negative trend and 4 countries show a positive trend. Santos et al. (Contribution 19) determined the potential use of the *Matricaria recutita* flower extract (MRFE) in combination with antineoplastic drugs to (1) enhance the antineoplastic effect, and (2) protect against the toxicity associated with the classical anticancer drugs. By in vitro and in vivo antitumor activity assays in a mouse tumor model, the results of this study suggest that MRFE 200 mg/kg/day can enhance the antitumor activity of 5-fluorouracil (5-FU), promoting the antineoplastic-induced reduction in body mass, while minimizing the toxicity of chemotherapy.

## Data Availability

Not applicable.
